# Subclinical wandering spleen in a cat with gastrointestinal lymphoma

**DOI:** 10.1002/ccr3.4891

**Published:** 2021-10-04

**Authors:** Masashi Yuki, Momoko Narita

**Affiliations:** ^1^ Yuki Animal Hospital Nagoya Aichi Japan

**Keywords:** cat, lymphoma, subclinical, wandering spleen

## Abstract

We accidentally detected a subclinical wandering spleen on preoperative ultrasonography in a cat with gastrointestinal lymphoma. If the spleen is not in the normal position, the wandering spleen should be considered.

## Graphical abstract

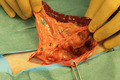

## 1 INTRODUCTION

The wandering spleen is a rare clinical entity characterized by splenic hypermobility caused by absent or abnormal laxity of the suspensory ligaments which fix the spleen in its normal position.^1^ These ligaments include the gastrosplenic, splenorenal, splenocolic, splenophrenic, and pancreaticosplenic ligaments.^2^ Ligamentous laxity can be acquired due to conditions such as splenomegaly or pregnancy; however, it is often congenital.^3^ Many cats with a wandering spleen have symptoms such as abdominal pain, but asymptomatic cases have also been reported.^4^ Treatment of the wandering spleen in humans involves the risk of torsion; hence, surgeries such as splenectomy or gastropexy are commonly performed.^5^

There have been several case reports on companion animals, such as dogs and cats, physical displacement caused by tissues other than the spleen, such as shrinkage and enlargement of organs around the spleen, rupture of the diaphragm and abdominal wall, hernia, and gastric torsion. Displacement due to twisting of the spleen is occasionally observed.^6^ However, there are no reports on the so‐called wandering spleens. To the best of our knowledge, this is the first report of a wandering spleen in a nonhuman mammal, a cat.

## 2 CASE HISTORY

A 12‐year‐old male castrated mixed‐breed cat was examined for a 5‐day history of lethargy, anorexia, and vomiting. The cat had no relevant medical history, and it received routine vaccinations. On physical examination, its weight was 3.2 kg, rectal temperature was 37.5℃, and heart rate was 160 beats/min. Its general appearance was quiet, alert, and responsive. The cat had a body condition score of 3 (on a scale of 1–5).

No abnormalities were found in the complete blood count. Biochemical analyses revealed abnormalities such as increased activities of alanine aminotransferase (99 U/L; reference interval [RI]: 22–84 U/L) and aspartate aminotransferase (82 U/L; RI: 18–51 U/L); increased concentrations of total protein (8.4 g/dl; RI: 5.7–7.8 g/dl), albumin (4.3 g/dl; RI: 2.3–3.5 g/dl), glucose (173 mg/dl; RI: 71–148 mg/dl), and urea nitrogen (45.9 mg/dl; RI: 17.6–32.8 mg/dl); and decreased concentrations of sodium (143 mEq/L; RI: 147–156 mEq/L), chloride (94 mEq/L; 107–120 mEq/L), and potassium (2.7 mEq/L; 3.4–4.6 mEq/L). The urinalysis results were unremarkable. Radiography of the thorax and abdomen revealed no abnormalities. Abdominal ultrasonography showed no apparent thickening of the intestinal wall but partial thickening of the muscularis (muscularis to submucosa ratio of 1:1) and swelling of the mesenteric lymph nodes (22 × 11 mm).

Inflammatory bowel disease and lymphoma were suspected, but at the request of the owner, symptomatic treatment was performed with subcutaneous infusions of Ringer's acetate solution (50 ml/kg), famotidine (1 mg/kg, PO, q24h), and metoclopramide (0.5 mg/kg, PO, q24h). The cat showed repeated improvement and worsening of clinical signs for 2 weeks. The results of the tests performed during this period showed that the activity of feline pancreas‐specific lipase (7.9 μg/L; RI <3.6 μg/L) had increased and the concentration of cobalamin (<150 ng/L; RI: 290–1000 ng/L) had decreased. The concentrations of total T4 (1.71 μg/L; RI: 0.6–3.9 μg/L) and folic acid (10.7 μg/L; RI: 9.7–21.6 μg/L) were normal. The tests were performed in a commercial veterinary medical laboratory.

**FIGURE 1:**
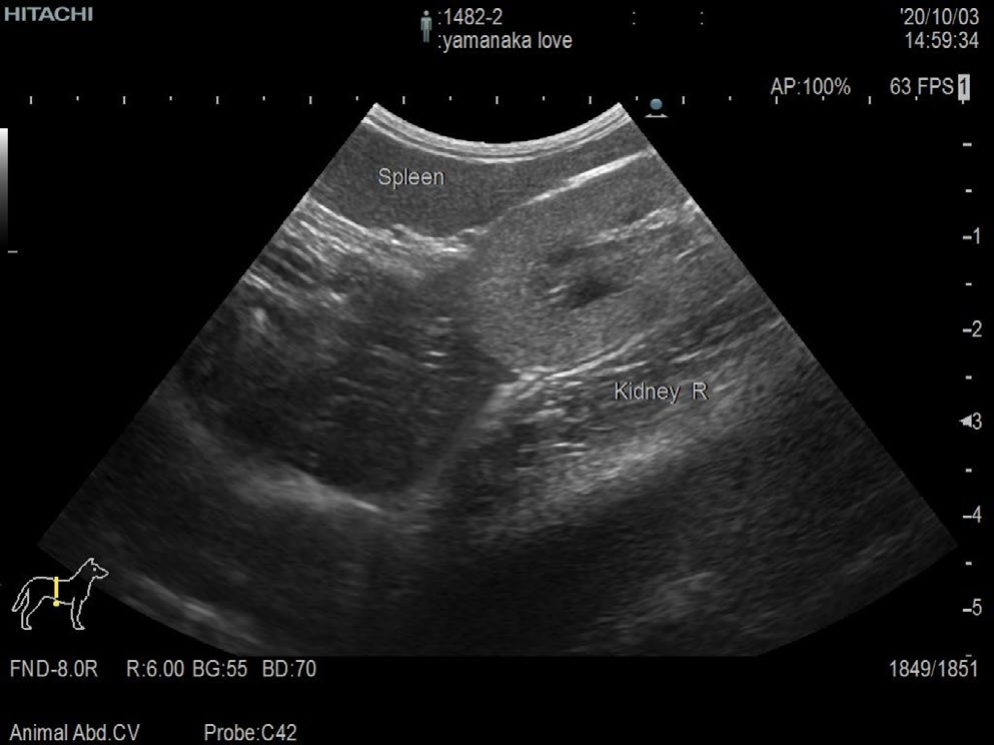
Abdominal ultrasonography findings on the day of surgery. The spleen is in contact with the right kidney and completely displaced to the right side of the abdominal cavity

**FIGURE 2:**
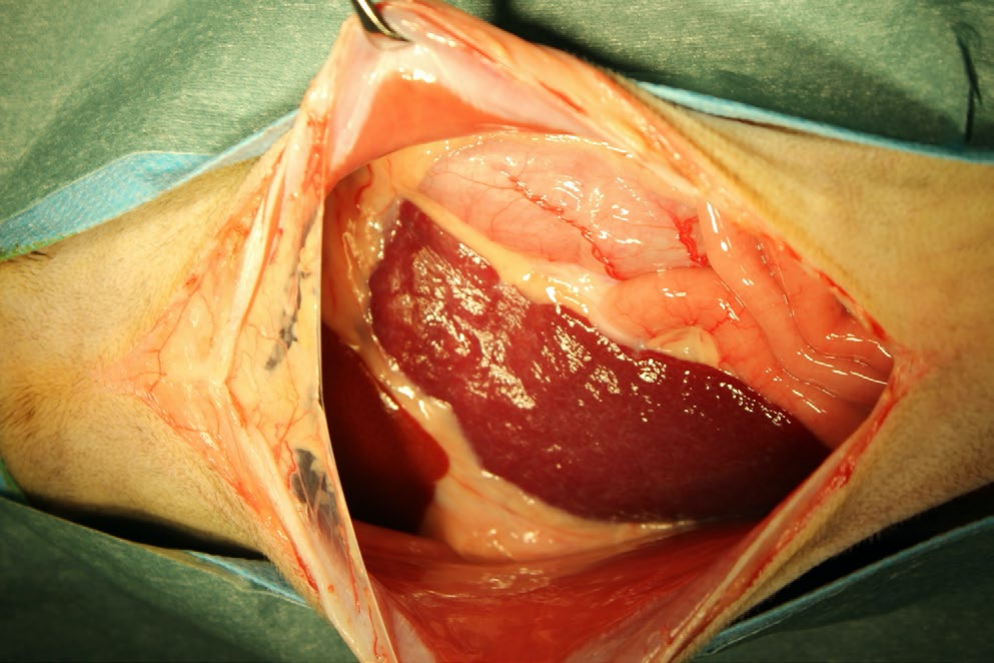
Macroscopic findings during laparotomy. The extremitas dorsalis of the spleen is completely displaced to the right side of the abdominal cavity. The left is the cranial side and the right is the caudal side

**FIGURE 3:**
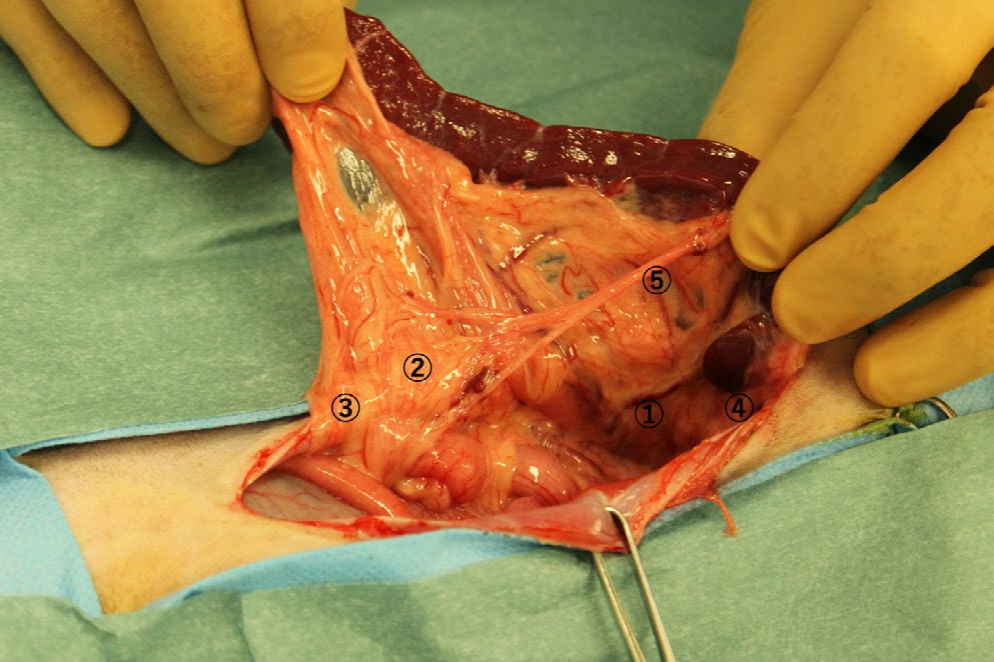
Blood vessels running through the spleen is normal. The gastrosplenic ①, splenorenal ②, splenocolic ③, splenophrenic ④, and pancreaticosplenic ⑤ ligaments supporting the spleen were clearly relaxed, and the spleen could be easily displaced. Notice that the surrounding organs are not lifted by the ligaments

On the 14th day, since there was no complete improvement in clinical signs, we decided to perform a full‐thickness biopsy of the intestinal tract by laparotomy. The cat was hospitalized, and an intravenous infusion of Ringer's acetate solution (10 ml/kg/h) was started. On the second day of hospitalization, all biochemical analysis items that were abnormal at the first visit were normal; only the serum amyloid A concentration (99.27 μg/dl; RI <2.5 μg/dl) was increased. Blood coagulation tests showed a low prothrombin time (8.0 s; RI: 9.3–10.7 s), a prolonged activated partial thromboplastin time (99.2 s; RI: 20.0–36.0 s), and an increased concentration of fibrinogen (283 mg/dl; RI: 120–240 mg/dl). The D‐dimer concentration (<0.6; RI: <0.6 μg/ml) was normal. Abdominal ultrasonography revealed no improvement in the thickening of the muscularis or swelling of the lymph nodes. Furthermore, the spleen, which was normal until the previous day, was displaced to the right side of the abdominal cavity (Figure 1). The cat was anesthetized, and biopsies of the intestinal tract and lymph nodes were performed. There were no obvious macroscopic abnormalities in the intestinal tract, but the mesenteric lymph nodes were swollen. A full‐thickness biopsy of the duodenum, jejunum, and ileum and a biopsy of the mesenteric lymph nodes were performed. The spleen size and color were normal (Figure 2). The blood vessels running through the spleen was normal, but abnormal relaxation of the gastrosplenic, splenorenal, splenocolic, splenophrenic, and pancreaticosplenic ligaments supporting the spleen were observed, and the spleen could be freely displaced (Figure 3).

Intravenous infusions of Ringer's acetate solution (10 ml/kg/h) and ceftriaxone sodium hydrate (20 mg/kg, SC, q12h) were administered for 3 days until discharge. During this period, the spleen was observed on ultrasonography, and there was no displacement from the normal position. Histopathological examination revealed a low‐grade gastrointestinal lymphoma. On the 25th day of hospitalization, anticancer therapy with prednisolone (2 mg/kg, PO, q24h) and chlorambucil (2 mg/cat, PO, every 2–3 days) was initiated. In addition, administration of cobalamin (250 μg/kg, SC, every week) was initiated for hypocobalaminemia. No displacement of the spleen was observed at this point either. Currently, 6 months have passed since the laparotomy, but no displacement of the spleen has been observed, and the gastrointestinal lymphoma is also in complete remission with tapering prednisolone (0.75 mg/kg, PO, q24h) and chlorambucil.

## 3 DISCUSSION

Many wandering spleens in humans are generally diagnosed by clinical symptoms, such as abdominal pain, ultrasonography, or computed tomography.^3^ In some cases, an asymptomatic wandering spleen is accidentally detected on abdominal ultrasonography.^4^ In the present case, the wandering spleen was accidentally detected on preoperative ultrasonography. Until the previous day of examination, the spleen was in the normal position, indicating that it was displaced from the left side to the right of the abdominal cavity in 1 day. A similar case has been reported in humans, wherein the spleen displaced to the right side of the abdominal cavity in 1 day, resulting in pancreatitis with abdominal pain and vomiting.^7^ The cat was hospitalized because of gastrointestinal symptoms, but no new clinical signs (eg, vomiting or abdominal pain) of displacement were observed. The lipase activity level, which suggests pancreatitis, was also high but before the displacement of spleen. Therefore, this case was considered to be a subclinical wandering spleen. Human wandering spleens are rare and reported cases are limited.^3^ To date, there have been no reports of wandering spleen in animals, and the incidence of wandering spleen in these animals may be lower than that in humans. If the spleen is not in the normal position, ectopic spleens, splenosis, and accessory spleens should be considered in the differential diagnosis.^3^, ^5^ Ectopic spleens have been reported in dogs and cats, but they are characterized by the presence of splenic tissue in the pancreas,^5^ which is inconsistent with this case. The spleen, in this case, was anatomically normal except for the position and was hence confirmed to be a wandering spleen.

In humans, the wandering spleen is often found in the pelvis, although sometimes it may remain in the left upper quadrant and simply have an abnormal rotation. Ligamentous laxity can be acquired due to conditions such as splenomegaly or pregnancy; however, it is often congenital.^3^ In the present case, the gastrosplenic, splenorenal, splenocolic, splenophrenic, and pancreaticosplenic ligaments supporting the spleen were clearly relaxed, and the spleen was completely displaced to the right side of the abdominal cavity. Based on the cat's past medical history, it was unlikely that the ligaments that support the spleen would be damaged. Furthermore, this cat was a male and hence, conditions such as pregnancy could not be considered. The spleen was also macroscopically normal, and splenomegaly was unlikely. These findings strongly suggest that this case had a congenital laxity or lack of ligaments that support the spleen.

There have been reports of generalized lymphoma in humans with a wandering spleen, wherein the wandering spleen was displaced in the pelvis, and the cause of displacement was unknown.^8^ This suggests that changes in the activity pattern or vascularization pattern in the spleen due to lymphoma may be a factor. In fact, lymphoma infiltration was observed in the spleen.^8^ The present case was diagnosed with low‐grade gastrointestinal lymphoma. The presence or absence of lymphoma infiltration is unknown because tissue biopsy of the spleen was not performed. The lymphoma achieved complete remission with chemotherapy, and at the same time, the wandering spleen has not recurred. From these facts, it is possible that the wandering spleen in this case was also due to lymphoma infiltration into the spleen. In addition, repeated vomiting due to lymphoma may have contributed to the wandering spleen. The hypothesis that the wandering spleen was caused by the addition of these factors to the congenital laxity or lack of ligaments that support the spleen was considered to be the most promising. The human wandering spleen is generally displaced to the pelvis, but in this case, it was displaced to the right side of the abdominal cavity. This difference is unknown, as this is the first time we have reported a case of wandering spleen in a companion animal, but it may be because cats are quadrupeds.

The treatment of wandering spleen in humans is generally surgical due to the risk of torsion. Splenectomy is selected for cases with splenic infarction, and splenopexy or gastropexy is selected for those without splenic infarction.^5^ Although conservative management is acceptable for an asymptomatic wandering spleen, elective splenopexy is also recommended because of the high incidence of torsion and trauma to the abnormally located spleen.^4^ In this case, the displacement of the spleen did not cause any clinical signs or gross changes; therefore, splenectomy was not performed, and the spleen was just returned to the normal position. No displacement of the spleen was observed thereafter, but considering the risks such as torsion of the spleen, it would have been better to perform splenopexy.

## 4 CONCLUSION

We accidentally detected a subclinical wandering spleen on preoperative ultrasonography in a cat with gastrointestinal lymphoma. The findings of our study strongly suggest that this case had a congenital laxity or lack of ligaments that support the spleen. To the best of our knowledge, this is the first report of a wandering spleen in a nonhuman mammal, a cat.

## CONFLICTS OF INTEREST

The authors declare no conflict of interest.

## AUTHOR CONTRIBUTIONS

MY contributed in writing—original draft preparation and supervision. MN is the primary clinician. Both authors have read and agreed to the published version of the manuscript.

## ETHICS APPROVAL

In Japan, there are no ethics committees available for private animal hospitals. Nevertheless, this study was conducted according to the ethical codes of the Japan Veterinary Medical Association. The samples obtained in this study were used after obtaining written consent from each dog owner.

## CONSENT

The samples obtained in this study were used after obtaining written consent from each dog owner.

## Data Availability

The authors declare that this paper does not include supplementary data.
